# Plasma metabolomic profiles of dementia: a prospective study of 110,655 participants in the UK Biobank

**DOI:** 10.1186/s12916-022-02449-3

**Published:** 2022-08-15

**Authors:** Xinyu Zhang, Wenyi Hu, Yueye Wang, Wei Wang, Huan Liao, Xiayin Zhang, Katerina V. Kiburg, Xianwen Shang, Gabriella Bulloch, Yu Huang, Xueli Zhang, Shulin Tang, Yijun Hu, Honghua Yu, Xiaohong Yang, Mingguang He, Zhuoting Zhu

**Affiliations:** 1 Department of Ophthalmology, Guangdong Academy of Medical Sciences, Guangdong Provincial People’s Hospital, Guangzhou, China; 2 Department of Ophthalmology, Shanghai General Hospital, Shanghai, China; 3 State Key Laboratory of Ophthalmology, Zhongshan Ophthalmic Center, Sun Yat-sen University, Guangzhou, China; 4 Neural Regeneration Group, Institute of Reconstructive Neurobiology, University of Bonn, Bonn, Germany; 5 Centre for Eye Research, University of Melbourne, East Melbourne, Victoria Australia; 6 Aier Institute of Refractive Surgery, Refractive Surgery Center, Guangzhou Aier Eye Hospital, Guangzhou, China; 7 Aier School of Ophthalmology, Central South University, Changsha, China

**Keywords:** Metabolites, Dementia, UK Biobank

## Abstract

**Background:**

Plasma metabolomic profile is disturbed in dementia patients, but previous studies have discordant conclusions.

**Methods:**

Circulating metabolomic data of 110,655 people in the UK Biobank study were measured with nuclear magnetic resonance technique, and incident dementia records were obtained from national health registers. The associations between plasma metabolites and dementia were estimated using Cox proportional hazard models. The 10-fold cross-validation elastic net regression models selected metabolites that predicted incident dementia, and a 10-year prediction model for dementia was constructed by multivariable logistic regression. The predictive values of the conventional risk model, the metabolites model, and the combined model were discriminated by comparison of area under the receiver operating characteristic curves (AUCs). Net reclassification improvement (NRI) was used to estimate the change of reclassification ability when adding metabolites into the conventional prediction model.

**Results:**

Amongst 110,655 participants, the mean (standard deviation) age was 56.5 (8.1) years, and 51 186 (46.3%) were male. A total of 1439 (13.0%) developed dementia during a median follow-up of 12.2 years (interquartile range: 11.5–12.9 years). A total of 38 metabolites, including lipids and lipoproteins, ketone bodies, glycolysis-related metabolites, and amino acids, were found to be significantly associated with incident dementia. Adding selected metabolites (*n*=24) to the conventional dementia risk prediction model significantly improved the prediction for incident dementia (AUC: 0.824 versus 0.817, *p* =0.042) and reclassification ability (NRI = 4.97%, *P* = 0.009) for identifying high risk groups.

**Conclusions:**

Our analysis identified various metabolomic biomarkers which were significantly associated with incident dementia. Metabolomic profiles also provided opportunities for dementia risk reclassification. These findings may help explain the biological mechanisms underlying dementia and improve dementia prediction.

**Supplementary Information:**

The online version contains supplementary material available at 10.1186/s12916-022-02449-3.

## Background

Dementia is a leading cause of disability in people over 65 years worldwide [1]. It is expected to affect over 131.5 million people and cost over a trillion dollars by 2050 [2]. As no effective treatments for dementia are currently available, early identification of patients at high risk is a public health priority in efforts to delay disease progression and alleviate the burden of disease on patients, policy makers, and healthcare providers [3]. Despite the plethora of research, current screening tools and prediction models are insufficiently accurate and are associated with high costs, invasive tests, and complex technicalities. Unfortunately, the early detection of dementia is a continually evolving field of research that frequently underdelivers to effectively target the needs and necessities of population-based screening.

Metabolomics comprehensively analyses small molecular metabolites in targeted tissues or biofluids, to indicate genetic, environmental, and pathological changes during disease development. Previous studies have revealed deranged metabolomic profiles in dementia patients, but further studies have provided conflicting conclusions [1, 4–6]. Such inconsistencies may be due to the small sample size, cross-sectional design, short follow-up time, limited adjustment for confounding factors, and different analytical chemistry techniques used to measure metabolomics in these studies. Furthermore, only few studies have investigated the additive value of plasma metabolites in dementia prediction, with inconsistent conclusions [7–13].

The UK Biobank Study is a prospective study that measured 249 metabolomic biomarkers in approximately 118,000 EDTA plasma samples using high-throughput nuclear magnetic resonance (NMR). Its large sample size, long-term follow-up, standardized platform of metabolomics profiling, and systematic ascertainment of incident dementia provide a unique opportunity to investigate the metabolomic profile of incident dementia and assess the additive values of metabolites for incident dementia.

## Methods

### Study participants

We studied participants in the prospective UK Biobank Study cohort that met the inclusion criteria of this study. The UK Biobank Study includes over 500,000 people of middle and old age that were recruited from 2006 to 2010 across the UK. Their baseline demographic, phenotypic, and genotypic characteristics were collected in 22 assessment centres at enrolment, and further collections were accumulated after follow-up intervals of 6 months to 3 years. Detailed protocols of the UK Biobank Study are described elsewhere [14].

**Fig. 1:**
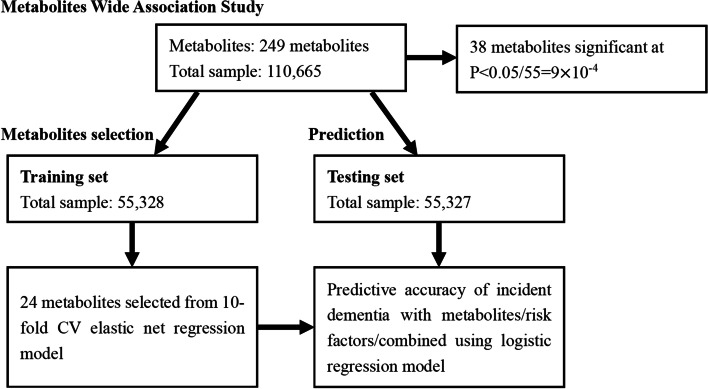
Data processing and analyses flow diagram of this study. Thirty-eight metabolites were significant following multiple testing in multi-variable cox proportional hazards models. For the development of a prediction model, participants were randomly assigned to the training and testing group for model development. After a 10-fold cross-validation test, 24 metabolites were assigned a nonzero coefficient in the elastic net regression model amongst the 249 included metabolites. Receiver operating characteristic (ROC) curve was created and area under curve (AUC) was calculated for predictive value comparison. Categorical net reclassification improvement (NRI) was calculated to investigate the reclassification ability

For the identification of metabolomic biomarkers associated with incident dementia, the present analyses included participants without prior dementia at baseline with available metabolomic data. A total of 110,730 participants had metabolites data, of which 75 participants with a history of dementia were excluded. Finally, 110,655 participants were included in the current analysis. To develop a prediction model for 10-year incident dementia risk, participants were randomly divided into a discovery group (*n* = 55,328) and a replication group (*n* = 55,327). The workflow of the analyses is presented in Fig. 1. Baseline characteristics of study participants in each dataset are described in Additional file 1: Table S1.

Ethics for the UK Biobank was approved by North West Multi-Centre Research Ethics Committee across the United Kingdom, and the Human Tissue Authority license was also approved for the UK Biobank. We gained access to the UK Biobank data through application. All participants submitted signed informed consent in written form. The Declarations of Helsinki were complied with throughout this study.

### Metabolite quantification

Detailed protocols on sample collection and metabolomic quantification are presented elsewhere [15–17]. In brief, EDTA plasma samples were collected at baseline recruitment (118,000 samples) and repeat assessment (5000 samples). Samples were prepared directly in 96-well plates by UK Biobank, with each plate containing a serum mimic as a quantification consistency monitor and a mixture of 2 low-molecular-weight metabolite as a technical reference. These samples were shipped to Nightingale Health's laboratories in Finland on dry ice and measured between June 2019 and April 2020. In the lab, samples were prepared with an automated liquid handler, automatically analysed with spectrometers and a robotic sample changer, and quantified with Nightingale Health’s proprietary software (Nightingale Health Biomarker quantification library 2020). Accredited quality control was done during the whole process to eliminate systemic and technical variance, and only samples and biomarkers that underwent the quality control process were stored in the UK Biobank dataset and used in our present study. Each sample included 168 metabolites in absolute level (mmol/L) spanning fatty acids, glycolysis metabolites, ketone bodies, amino acids, lipids, and lipoproteins, and 81 in ratio measurement.

### Ascertainment of dementia

Dementia incidence data were collected through hospital in-patient admission records and death registries. The identification of dementia was based on the International Classification of Diseases (ICD) code, including 290.0–290.4, 294.1, 331.0–331.2, and 331.5 in ICD-9 and A81.0, F00, F01, F02, F03, F05.1, F10.6, G30, G31.0, G31.1, and G31.8 in ICD-10, covering Alzheimer's Disease dementia, vascular dementia, and dementias of other causes. The follow-up period was defined from baseline to the earliest one amongst the incident dementia date, lost-of-follow-up date, death date, or the last date of data update, which was April 28, 2021.

### Traditional risk factors

Age [18], gender [18], education level [18], systolic pressure [19], anti-hypertension treatment [20], diabetes mellitus [21], smoking status [22], history of stroke [23], history of coronary heart disease [24], and APOE ε4 allele were established risk factors of dementia [25], and therefore were used as covariates in Cox regression analysis and constituted conventional prediction model for dementia. Age (UKB Field 21022) and systolic pressure (UKB Filed 4080) were continuous variables and gender (UKB Field 31, female or male), education level (UKB Field 6138, college/university degree or others), anti-hypertension treatment (UKB Field 6153, no or yes), diabetes mellitus (no or yes, including UKB Field 2443, doctor-diagnosed diabetes, UKB Field 6153, insulin treatment, UKB Field 20003, diabetes-related medication and UKB Field 30750, plasma HbA1c level of or over 48 mmol/mol), smoking status (UKB Field 20116-0.0, never or former/current), history of stroke (UKB Field 4056, no or yes), history of coronary heart disease (UKB Field 3627,3894, no or yes), and APOE ε4 carrier (no or yes) were redefined as categorical variables.

### Statistical analyses

Continuous variables were described with mean (standard deviation, SD) or median (interquartile range, IQR), and categorical variables were described with number and percentage. The values of all metabolites were first transformed using natural logarithmic transformation (ln[x+1]) and then *Z*-transformed. Our present study included two separate analyses (Fig. 1). In the first analysis, the associations between metabolites and dementia were estimated using cox proportional-hazard models, with confounders including age, sex, education level, systolic pressure, anti-hypertension treatment, diabetes mellitus, smoking status, history of stroke, history of coronary heart disease, APOE ε4 allele adjusted. A P value less than 0.05 was set as nominal significance. The corrected P-value was estimated through a principal component analysis developed by Gao et al. [26]. Strong correlations were considered, and 55 parameters explained 99.5% of metabolites variations. Therefore, 55 independent tests were conducted for correlation ascertainment, and *P* value significance was set at 9×10^−4^ (0.05/55) or less. β coefficients of adjusted hazard ratio (HR), 95% coefficient interval (CI) and P values of all 249 metabolites in the cox model are presented in Additional file 1: Table S2.

In the second analysis, participants were randomly assigned to a training set (*n*=55,328) and a testing set (*n*=55,327) to develop and validate the 10-year incident dementia risk prediction model. We used elastic net regularized logistic regression models to select metabolomic predictors. The elastic net regularized logistic regression model is a straightforward supervised machine learning algorithm combining least absolute shrinkage and selection operator (LASSO) regression and Ridge regression with a good probabilistic interpretation of variables suitable for disease prediction. The LASSO penalty selected variables by reducing the absolute value of weight, while the Ridge penalty further reducing the extremities of weights. Details of the model have been described elsewhere [27]. Two tuning parameters for elastic net regression model were used, including *α* (representing the weight of the penalty) and *λ* (representing the complexity of the penalty). Of note, *α* controls the balance between LASSO and Ridge, with α(1) corresponding to the lasso (the default estimator) and *α*(0) corresponding to ridge regression [28]. To achieve the sparsity of the model and select core metabolomic predictors, we tested α of 0.5, 0.75, and 1. We then used 10-fold cross-validation to select optimal *λ* and *β* coefficients for elastic net regression models in seek of minimum to minimize cross-validation prediction error, and order to achieve optimal robustness of the model. Each combination of *α* and *λ* on a two-dimensional grid underwent 10-fold cross-validation for elastic net logistic regression to compute cross-validation prediction error and assign *β* coefficient for each metabolite to achieve the minimum cross-validation function. In the final model, *α*=1 and *λ*=0.0003648 were selected by cross-validation. Minimum cross-validation mean deviance was 0.0887588. 24 metabolites were given nonzero coefficient (Additional file 1: Table S3). In the testing set, we applied three logistic regression models to estimate the predictive values of different parameters. Model 1 included conventional risk factors, including age, gender, education level, systolic pressure, anti-hypertension treatment, diabetes mellitus, smoking status, history of stroke, history of coronary heart disease, and APOE ε4 allele; Model 2 included selected metabolomic biomarkers; and Model 3 included combined conventional risk factors and selected metabolomic biomarkers. Coefficients (95% CI) and *P* value for each exposure parameter are presented in Additional file 2: Table S4. The predictive value of the selected 24 metabolites was assessed through two methods. Firstly, the receiver operating characteristic (ROC) curve was constructed and the areas under the curves (AUCs) were compared amongst different models. Secondly, using > 5% as the threshold for the group at high risk to develop dementia in 10 years [29], categorical net reclassification improvement (NRI) was estimated for the added value of selected metabolomic biomarkers for risk stratification over conventional risk factors.

All analyses were performed using Stata version 13 (Stata Corp) and R (version 3.3.1, R Project for Statistical Computing, Vienna, Austria).

## Results

### Baseline characteristics of the study participants

Our analysis included 110,655 participants without dementia at baseline, with an average (SD) age of 56.5 (8.1), of which 59,469 (53.7%) were female. After a median follow-up of 12.2 years (IQR: 11.5–12.9 years), 1439 (1.30%) participants developed dementia. Baseline characteristics of all participants stratified by incident dementia are summarized in Table 1. Participants with incident dementia were often older, male, APOE ε4 carriers, with lower education, higher systolic pressures, a history of diabetes mellitus, anti-hypertensive medication use, former or current smokers, and had a history of stroke or coronary heart disease.

**Table 1:** Baseline characteristics of study participants in the prospective study of dementia

Baseline Characteristics	Overall (***N***= 110,655)	Individuals with incident dementia (***n***=1439)	Individuals without incident dementia (***n***=109,216)	***P*** value
Age, mean (SD), years	56.5 (8.10)	64.2 (4.88)	56.4 (8.08)	**<0.001**
Gender, No. (%)				**<0.001**
Female	59,469 (53.7)	652 (45.3)	58,817 (53.9)	
Male	51,186 (46.3)	787 (54.7)	50,399 (46.1)	
Education level, No. (%)				**<0.001**
College or university degree	35,744 (32.3)	301 (20.9)	35,443 (32.5)	
Others	74,911 (67.7)	1138 (79.1)	73,773 (67.5)	
Systolic pressure, mean (SD), mmHg	137 (18.5)	143 (19.3)	138 (18.5)	**<0.001**
Anti-hypertension treatment, No. (%)				**<0.001**
No	100,148 (90.5)	1211 (84.2)	98,937 (90.6)	
Yes	10,507 (9.50)	228 (15.8)	10,279 (9.41)	
Diabetes mellitus, No. (%)				**<0.001**
No	103,950 (93.9)	1207 (83.9)	102,743 (94.1)	
Yes	6705 (6.06)	232 (16.1)	6473 (5.93)	
Smoking status, No. (%)				**<0.001**
Never	60,195 (54.7)	666 (46.6)	59,529 (54.8)	
Former/current	49,896 (45.3)	762 (53.4)	49,134 (45.2)	
History of stroke, No. (%)				**<0.001**
No	109,108 (98.6)	1361 (94.6)	107,747 (98.7)	
Yes	1547 (1.40)	78 (5.42)	1469 (1.35)	
History of coronary heart disease, No. (%)				**<0.001**
No	106,067 (95.9)	1267 (88.1)	104,800 (96.0)	
Yes	4588 (4.15)	172 (12.0)	4416 (4.04)	
APOE ε4 carrier, No. (%)				**<0.001**
No	83,454 (75.8)	795 (55.6)	82,649 (76.0)	
Yes	26,675 (24.2)	635 (44.4)	26,040 (24.0)	

*SD* standard deviation, *No.* number

### Associations of baseline circulating metabolites with incident dementia

**Fig. 2:**
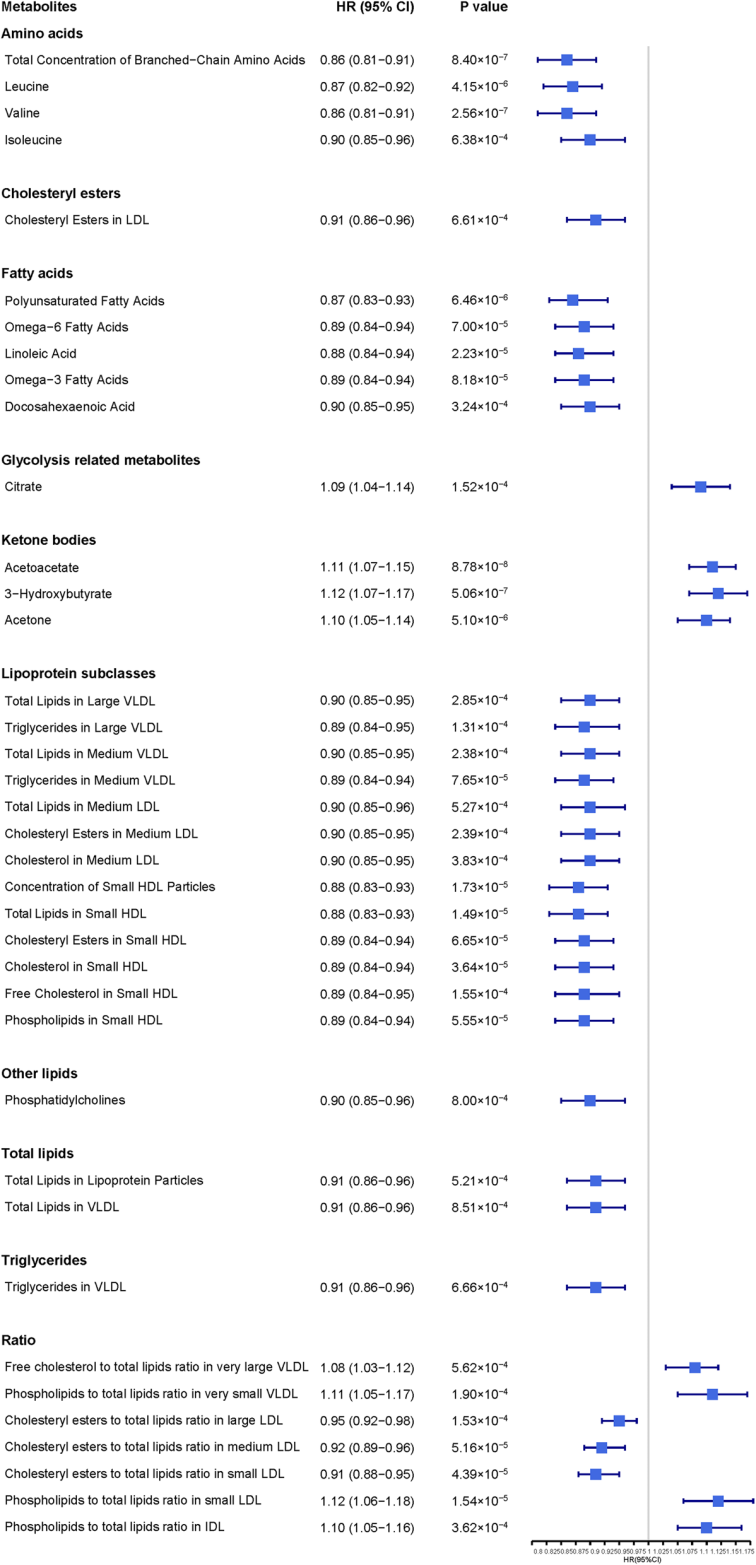
Adjusted HR (95% CI) of incident dementia for metabolites after multiple testing. Hazard ratios (HR) are per 1 standard deviation (SD) higher of Z-transformed metabolic marker and are adjusted for age, gender, education level, systolic pressure, anti-hypertension treatment, diabetes mellitus, smoking status, history of stroke, history of coronary heart disease, and APOE ε4 allele. CI, confidence interval; LDL, low-density lipoprotein; HDL, high-density lipoprotein; VLDL, very-low-density lipoprotein; IDL, intermediate-density lipoprotein

Amongst the 249 metabolomic biomarkers tested by UK Biobank Study with NMR technique, after controlling for multiple testing, 38 metabolites remained significantly associated with dementia incidence (Fig. 2). These metabolites included amino acids, fatty acids, glycolysis-related metabolites, ketone bodies, and lipids and lipoproteins categories. Only eight metabolomic biomarkers, including citrate (HR=1.09 [95% CI: 1.04–1.14], *P*=1.52×10^−4^), three ketone bodies (e.g., HR_Acetoacetate_=1.11 [95%CI: 1.07–1.15], *P*=8.78×10^−8^), phospholipids to total lipids ratio in intermediate-density lipoproteins (IDL), small low-density lipoproteins (LDL), and very small very-low-density lipoproteins (VLDL) (e.g., HR_s-LDL-PL-%_=1.12 [95% CI: 1.06–1.18], *P*=1.54×10^−5^), and free cholesterol to total lipids ratio in very large VLDL (HR_VL-VLDL-FC-%_=1.08 [95% CI: 1.03–1.12], *P*=5.62×10^−4^), were positively associated with dementia, while 30 other metabolomic biomarkers from amino acids, fatty acids, lipids and lipoprotein subclasses, were inversely associated with the incident dementia.

### Metabolite selection, prediction, and reclassification of the incident possibility of dementia

**Fig. 3:**
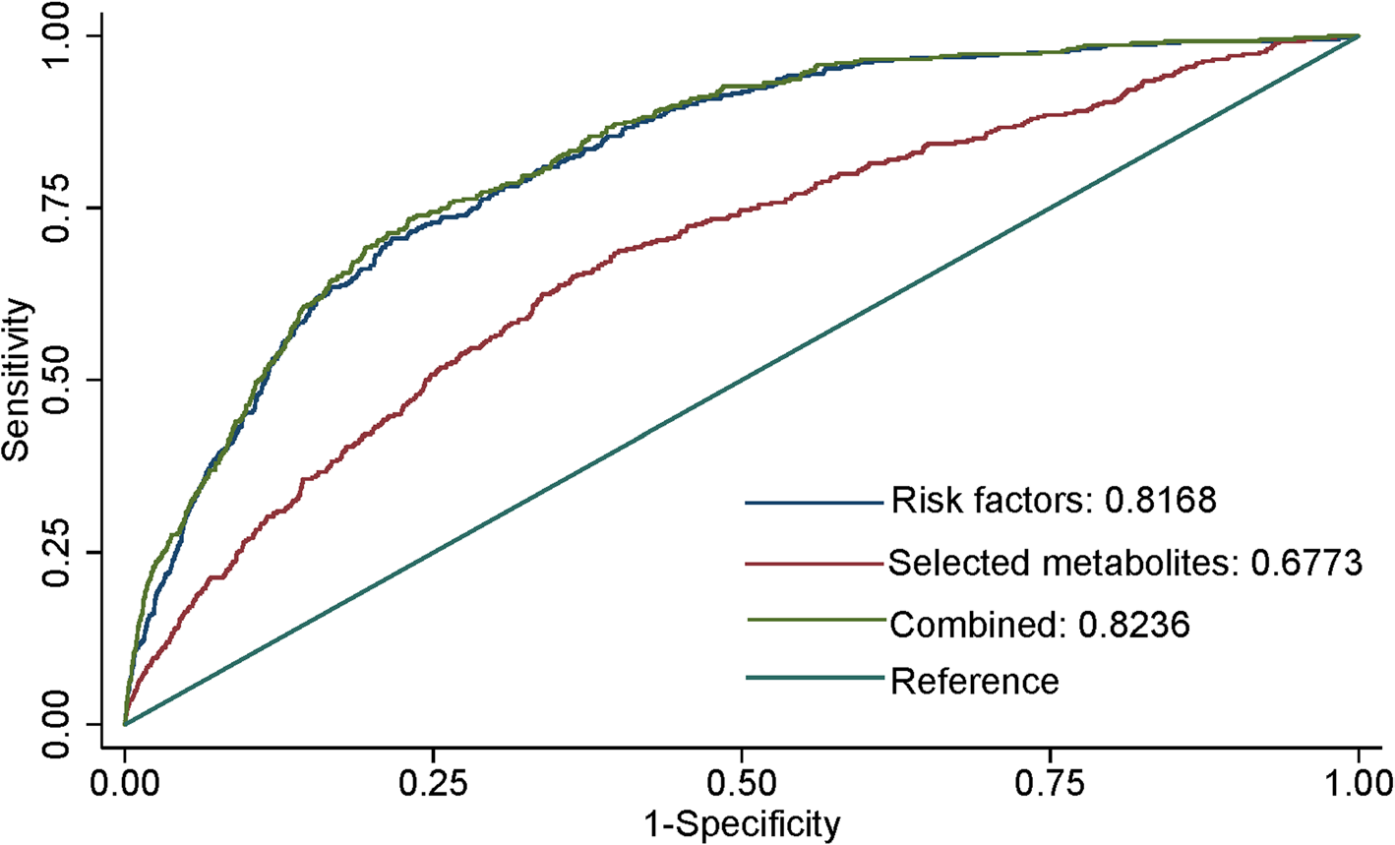
ROC and AUC analysis of incident dementia prediction model development and predictive value comparison. An elastic net regression model based on lasso penalty was used for dementia prediction. After 10-fold cross-validation, 24 of 249 metabolites were selected for the dementia prediction model. Xb1 curve used conventional risk factors as input signals, while the Xb2 curve was for 24 selected metabolites and Xb3 was for conventional risk factors and 24 selected metabolites. There was no clinically significant difference (*P* = 0.042) found between the AUC of Xb1 and Xb3. Conventional risk factors included age, gender, education level, systolic pressure, anti-hypertension treatment, diabetes mellitus, smoking status, history of stroke, history of coronary heart disease, and APOE ε4 allele. ROC, receiver operating characteristic; AUC, area under curve

Elastic net regularized logistic regression analysis identified metabolites for the construction of a dementia prediction model, and 24 metabolites (Additional file 1: Table S3) were selected for inclusion in the training set. The prediction ability of these metabolites (Xb2) was worse than the conventional prediction model (Xb1) (AUC: 0.677 versus 0.817), although the addition of these metabolites to the conventional risk factors-based model (Xb3) improved the prediction precision (AUC: 0.824 versus 0.817, *p*=0.042) (Fig. 3). Categorical net reclassification improvement analysis showed significant benefit in reclassification ability through the addition of metabolomic biomarkers into the conventional prediction model (NRI=4.97%, SE=0.009, *p*<0.001) (Table 2).

**Table 2:** Net reclassification improvement of adding 24 metabolites into a conventional risk prediction model

	Conventional prediction model	Updated model	
Incident dementia in 10 years	Risk (%)	<5	>5
Yes	<5	668	48
	>5	6	76
No	<5	101,185	654
	>5	353	823

Values are *n*. Patients in the upper-right cell were rightfully up-reclassified (*n*=48, and 6 patients were mistakenly down-reclassified), indicating an improved sensitivity. Patients in the lower-left cell were rightfully down-reclassified (*n*=353, and 654 patients mistakenly up-reclassified), indicating an undermined specificity. NRI (SE) = 0.0497 (0.00921), *P* value <0.001·

## Discussion

Using data from over 100,000 plasma samples from UK Biobank, we discovered that 38 serum metabolites including amino acids, ketone bodies, glycolysis metabolites, lipids, and lipoproteins were significantly associated with an increased risk of incident dementia. Our findings suggest the addition of metabolites into dementia risk stratification models could improve its prediction and reclassification for at-risk individuals. These metabolites also further insights to biological mechanisms which may underly dementia, and may assist in the prediction of dementia.

Our results showed branched-chain amino acids (BCAAs) are negatively associated with an elevated risk of incident dementia, which is consistent with several previous studies [1, 30]. It is suggested circulatory BCAAs may pass the blood-brain barrier (BBB) [1] and participate in the synthesis of key neurotransmitters, proteins, and energy [31], which may exert neuroprotective benefits for the ageing brain. A recent randomized clinical trial also suggested dietary BCAA supplements improved cognitive function in middle-aged and older adults [32]. As such, this study supports the role of BCAAs for dementia prevention, and this should be further investigated as a possible modifiable factor for delaying dementia onset.

Our analysis also implicated ketone bodies and citrate as metabolites positively associated with incident dementia. Elevation of plasma ketone bodies infer a switch from glucose-dependent substrates as energy, which occurs in low carbohydrate diets, disrupted glucose uptake disorders like diabetes, or during long periods of fasting. Ketone bodies have also been implicated in cognitive impairment and Alzheimer’s dementia (AD) brains which were exposed to long-term glucose uptake and utilization disruption [33, 34]. This association is further explained by recent evidence suggesting the utilization of ketone bodies in the brain largely relies on their plasma concentration [35] and transportation capacity across the BBB [36]. Our association with peripheral citrate is concordant with FA Leeuw et al. whom observed higher levels of plasma citrate were associated with brain and hippocampal atrophy, and white matter hyperintensity, which are known neurological changes associated with AD and dementia [37]. Furthermore, these metabolic disturbances are replicated in animal AD model brains [33, 38, 39]. Although our findings are consistent with previous studies, the exact mechanisms underlying these associations are lacking. We suggest future research to concentrate on laboratory and clinical studies to further define and elucidate the roles of ketones and citrate in dementia.

Strong negative correlations were observed between small high-density lipoproteins (HDL), its lipid constituents (including total lipids, cholesteryl esters, cholesterol, free cholesterol, and phospholipids) and incident dementia in the present analysis. Similar findings have been replicated by previous studies although an adequate explanation on these observations is yet to be fully realized [37, 40, 41]. Of note, Martinez et al. recently showed small HDL was the only lipoprotein that could pass the BBB, which implies it plays a role in the balance and distribution of fats within the brain [42]. Potentially, HDL exerts its neuroprotective effects through the redistribution of lipids which may affect neuronal membrane composition. This may have reverberating effects on *β* deposition and p-tau accumulation, and may protect neurons against oxidation and inflammation, thereby preserving vascular and synaptic function [40, 42, 43].

Total lipids, cholesteryl esters, cholesterol, triglycerides in VLDL and LDL subclasses, and polyunsaturated fatty acids (PUFA) were inversely associated with future risk of dementia, while free cholesterol concentration in very large VLDL was associated with an increased risk of dementia. These findings are aligned with some previous studies [1, 40, 44, 45], although a body of evidence has suggested conflicting results [46–48]. Of note, LDL-C is a conventional risk factor in cardiovascular and metabolic diseases, although its role in dementia has been historically conflicting [48, 49]. Furthermore, the different roles of lipoprotein subclasses may be attributed to their ability to cross the BBB, which makes the lipid environment in the brain very different from the periphery [37]. Nonetheless, several hypotheses may explain our findings, including the presumed role of triglycerides for interfering with peripheral Aβ transportation and facilitating PUFA absorption [50]. PUFAs are not only an anti-oxidative and anti-inflammatory regulator through modulating pro-inflammatory cytokines and decreasing microglial inflammatory activation of Aβ [50], but also are an essential component of neuronal membranes and can pass the BBB to participate in brain development [51], so theoretically their presence in serum should indicate their active synthesis and transportation which would be beneficial for the brain. The links between VLDL and LDL with dementia are less straightforward, as these lipoproteins cannot pass the BBB. Further studies are needed to investigate their mechanisms and tie in their association with dementia pathogenesis.

Furthermore, we successfully identified candidate metabolites, whose addition to the conventional model could significantly improve the accuracy of dementia prediction and reclassification of risk group, thus might improve the sensitivity of identifying patients in their prodromal phase. Such findings suggest the potential clinical use of metabolomic biomarkers as complementary information for early and population-based detection of dementia. However, though our model showed statistically significant improvement through the addition of metabolites, caution is still required when interpreting clinical implications as the absolute increase was not substantial. This was possible because conventional risk factors for dementia, e.g., diabetes and hypertension, already accounted for some of the metabolic changes in dementia pathogenesis.

Our study had several strengths, including the use of over 100,000 samples over a 14-year follow-up duration, the adjustment for confounding factors, and a homogeneous platform to analyse metabolites using the NMR technique [52]. Some limitations must also be acknowledged. Firstly, the participants were largely Caucasian and from developed countries with good socioeconomic standing [53] which may limit the generalizability of our results to other ethnicities and geographic backgrounds. Secondly, our longitudinal associations do not imply causality, hence more research is needed to confirm our findings. Thirdly, though the identification of clinical dementia from the UK electronic healthcare dataset guaranteed the specificity of our model, the omission of subclinical dementia may undermine its sensitivity [54, 55]. Furthermore, the paper did not define the effect of metabolites on predicting various subtypes of dementia. Fourthly, as we used peripheral metabolomic data rather than CNS metabolites, these results may not reflect the brain microenvironment and should be interpreted with caution. Lastly, we cannot exclude residual confounders.

## Conclusions

By use of a novel study design to investigate a prospective cohort of 110,655 participants over 14 years, several metabolomic biomarkers were found significantly associated with dementia incidence. The metabolites identified in this study may supplement future hypotheses explaining the complex interplay between energy and lipid metabolism, and the development of dementia. These metabolites improved risk stratification when added to conventional risk factor models, and this study provides an opportunity for considering the improvement of screening tools to better identify at-risk populations. Our findings also further discuss biological mechanisms underlying dementia and potentially facilitate the prediction, prevention, and treatment of dementia. Molecular and genetic studies were needed to determine the exact pathways mediating our observed associations, and clinical studies are needed to prove these metabolites can improve screening and prediction of dementia.

## Supplementary Information

**Additional file 1: Table S1**. Baseline characteristics of study participants stratified by discovery or replication dataset. **Table S2**. β coefficient of adjusted HR (95% CI) and *P* values of Incident Dementia for All 249 Metabolites in Cox Proportional Hazards Model. **Table S3**. Coefficients of selected 24 metabolites in elastic net regularized logistic regression model after 10-fold cross validation, their coefficients in e-net regression model in training dataset, and their VIFs in testing dataset.

**Additional file 2: Table S4**. β coefficients (95% CI) and *P* values of conventional risk factors and metabolites in the multi-variable logistic model for 10-year incident dementia prediction.

## Data Availability

The dataset analysed during the present study is available in the UK Biobank (https://www.ukbiobank.ac.uk/). These data are not publicly available because they were analysed under licence, but they are available from the corresponding author on reasonable request and with permission of UK Biobank.

## Abbreviations

AUC: Area under the receiver operating characteristic curves
AD: Alzheimer’s dementia
BBB: Blood-brain barrier
BCAA: Branched-chain amino acids
CI: Coefficient interval
HDL: High-density lipoproteins
HR: Hazard ratio
ICD: International Classification of Diseases
IDL: Intermediate-density lipoproteins
IQR: Interquartile range
LASSO: Least absolute shrinkage and selection operator
LDL: Low-density lipoproteins
NMR: Nuclear magnetic resonance
NRI: Net reclassification improvement
PUFA: Polyunsaturated fatty acids
ROC: Receiver operating characteristic
SD: Standard deviation
UKB: The United Kingdom Biobank
VLDL: Very-low-density lipoproteins
