# Supplementary material for: Plasma metabolomic profiles of dementia: a prospective study of 110,655 participants in the UK Biobank
Source: BMC Med. 2022 Aug 15;20:252. doi: 10.1186/s12916-022-02449-3 (PMC9377110; doi:10.1186/s12916-022-02449-3)
Supplement: Supplementary file 1 — Additional file 1: Table S1. Baseline characteristics of study participants stratified by discovery or replication dataset. Table S2. β coefficient of adjusted HR (95% CI) and P values of Incident Dementia for All 249 Metabolites in Cox Proportional Hazards Model. Table S3. Coefficients of selected 24 metabolites in elastic net regularized logistic regression model after 10-fold cross validation, their coefficients in e-net regression model in training dataset, and their VIFs in testing dataset. [file 12916_2022_2449_MOESM1_ESM.docx]

Supplemental Table 1: Baseline characteristics of study participants stratified by discovery or replication dataset

| Baseline Characteristics | Overall  (N= 110,655) | Individuals in discovery dataset  (n=55,328) | Individuals in replication dataset  (n=55,327) | P value |
| --- | --- | --- | --- | --- |
| Age, mean (SD), yrs | 56.5(8.10) | 56.5(8.11) | 56.5(8.09) | 0.752 |
| Gender, No. (%) |  |  |  | 0.878 |
| Female | 59,469(53.7) | 29,722(53.7) | 29,747(53.8) |  |
| Male | 51,186(46.3) | 25,606(46.3) | 25,580(46.2) |  |
| Education level, No. (%) |  |  |  | 0.776 |
| College or university degree | 35,744(32.3) | 17,850(32.3) | 17,894(32.3) |  |
| Others | 74,911(67.7) | 37,478(67.7) | 37,433(67.7) |  |
| Systolic pressure, mean (SD), mmHg | 137(18.5) | 138(18.6) | 138(18.5) | 0.720 |
| Anti-hypertension treatment, No. (%) |  |  |  | 0.179 |
| No | 100,148(90.5) | 50,140(90.6) | 50,008(90.4) |  |
| Yes | 10,507(9.50) | 5,188(9.38) | 5,319(9.61) |  |
| Diabetes mellitus, No. (%) |  |  |  | 0.252 |
| No | 103,950(93.9) | 51,930(93.9) | 52,020(94.0) |  |
| Yes | 6,705(6.06) | 3,398(6.14) | 3,307(5.98) |  |
| Smoking status, No. (%) |  |  |  | 0.589 |
| Never | 60,195(54.7) | 30,143(54.8) | 30,052(54.6) |  |
| Former/current | 49,896(45.3) | 24,904(45.2) | 24,992(45.4) |  |
| History of stroke, No. (%) |  |  |  | 0.069 |
| No | 109,108(98.6) | 54,519(98.5) | 54,589(98.7) |  |
| Yes | 1,547(1.40) | 809(1.46) | 738(1.33) |  |
| History of coronary heart disease, No. (%) |  |  |  | 0.546 |
| No | 106,067(95.9) | 53,054(95.9) | 53,013(95.8) |  |
| Yes | 4,588(4.15) | 2,274(4.11) | 2,314(4.18) |  |
| APOE ε4 carrier, No. (%) |  |  |  | 0.786 |
| No | 83,454(75.8) | 41,702(75.7) | 41,752(75.8) |  |
| Yes | 26,675(24.2) | 13,355(24.3) | 13,320(24.2) |  |
| Incident dementia events, No. (%) | 1439(1.30) | 714(1.29) | 725(1.31) | 0.395 |
| Survival time, mean (SD) years | 11.9(1.75) | 11.9(1.74) | 11.9(1.76) | 0.177 |

SD=Standard Deviation; No.=Number.

Supplemental Table 2. β coefficient of adjusted HR (95% CI) and P values of Incident Dementia for All 249 Metabolites in Cox Proportional Hazards Model.

| Metabolites |  | β (95% CI) | P-value |
| --- | --- | --- | --- |
| Cholesterol |  |  |  |
| Total Cholesterol |  | -0.085(-0.143, -0.028) | **3.69×10^-3^** |
| Total Cholesterol Minus HDL-C |  | -0.088(-0.144, -0.031) | **2.27×10^-3^** |
| Remnant Cholesterol (Non-HDL, Non-LDL -Cholesterol) |  | -0.076(-0.133, -0.020) | **8.33×10^-3^** |
| VLDL Cholesterol |  | -0.073(-0.129, -0.016) | **1.18×10^-2^** |
| Clinical LDL Cholesterol |  | -0.081(-0.137, -0.025) | **4.40×10^-3^** |
| LDL Cholesterol |  | -0.094(-0.150, -0.037) | **1.11×10^-3^** |
| HDL Cholesterol |  | -0.021(-0.082, 0.041) | 5.11×10^-1^ |
| Triglycerides |  |  |  |
| Total Triglycerides |  | -0.093(-0.150, -0.036) | **1.37×10^-3^** |
| Triglycerides in VLDL |  | -0.099(-0.156, -0.042) | **6.65×10^-4^** |
| Triglycerides in LDL |  | -0.034(-0.091, 0.022) | 2.32×10^-1^ |
| Triglycerides in HDL |  | -0.070(-0.128, -0.013) | **1.62×10^-2^** |
| Phospholipids |  |  |  |
| Total Phospholipids in Lipoprotein Particles |  | -0.090(-0.148, -0.031) | **2.78×10^-3^** |
| Phospholipids in VLDL |  | -0.081(-0.137, -0.024) | **5.36×10^-3^** |
| Phospholipids in LDL |  | -0.084(-0.142, -0.027) | **3.85×10^-3^** |
| Phospholipids in HDL |  | -0.039(-0.100, 0.022) | 2.10×10^-1^ |
| Cholesteryl esters |  |  |  |
| Total Esterified Cholesterol |  | -0.085(-0.143, -0.027) | **3.83×10^-3^** |
| Cholesteryl Esters in VLDL |  | -0.063(-0.120, -0.006) | **3.04×10^-2^** |
| Cholesteryl Esters in LDL |  | -0.098(-0.155, -0.042) | **6.60×10^-4^** |
| Cholesteryl Esters in HDL |  | -0.020(-0.080, 0.041) | 5.28×10^-1^ |
| Free cholesterol |  |  |  |
| Total Free Cholesterol |  | -0.084(-0.142, -0.026) | **4.67×10^-3^** |
| Free Cholesterol in VLDL |  | -0.079(-0.136, -0.022) | **6.45×10^-3^** |
| Free Cholesterol in LDL |  | -0.077(-0.134, -0.019) | **8.69×10^-3^** |
| Free Cholesterol in HDL |  | -0.018(-0.080, 0.044) | 5.71×10^-1^ |
| Total lipids |  |  |  |
| Total Lipids in Lipoprotein Particles |  | -0.099(-0.155, -0.043) | **5.20×10^-4^** |
| Total Lipids in VLDL |  | -0.095(-0.151, -0.039) | **8.50×10^-4^** |
| Total Lipids in LDL |  | -0.091(-0.148, -0.035) | **1.43×10^-3^** |
| Total Lipids in HDL |  | -0.037(-0.099, 0.0241) | 2.33×10^-1^ |
| Lipoprotein particle concentrations |  |  |  |
| Total Concentration of Lipoprotein Particles |  | -0.093(-0.154, -0.033) | **2.55×10^-3^** |
| Concentration of VLDL Particles |  | -0.071(-0.129, -0.014) | **1.45×10^-2^** |
| Concentration of LDL Particles |  | -0.068(-0.126, -0.010) | **2.08×10^-2^** |
| Concentration of HDL Particles |  | -0.084(-0.145, -0.024) | **6.02×10^-3^** |
| Lipoprotein particle sizes |  |  |  |
| Average Diameter for VLDL Particles |  | -0.037(-0.066, -0.008) | **1.24×10^-2^** |
| Average Diameter for LDL Particles |  | 0.053(-0.003, 0.109) | **6.49×10^-2^** |
| Average Diameter for HDL Particles |  | 0.092(0.000, 0.183) | **4.92×10^-2^** |
| Other lipids |  |  |  |
| Phosphoglycerides |  | -0.092(-0.152, -0.033) | **2.20×10^-3^** |
| Total Cholines |  | -0.089(-0.149, -0.030) | **3.30×10^-3^** |
| Phosphatidylcholines |  | -0.102(-0.162, -0.042) | **7.99×10^-4^** |
| Sphingomyelins |  | -0.028(-0.088, 0.032) | 3.55×10^-1^ |
| Apolipoproteins |  |  |  |
| Apolipoprotein B |  | -0.070(-0.127, -0.013) | **1.59×10^-2^** |
| Apolipoprotein A1 |  | -0.060(-0.121, 0.000) | **5.11×10^-2^** |
| Fatty acids |  |  |  |
| Total Fatty Acids |  | -0.087(-0.143, -0.031) | **2.28×10^-3^** |
| Degree of Unsaturation |  | -0.055(-0.109, -0.001) | **4.71×10^-2^** |
| Omega-3 Fatty Acids |  | -0.115(-0.172, -0.058) | **8.18×10^-5^** |
| Omega-6 Fatty Acids |  | -0.118(-0.176, -0.060) | **7.00×10^-5^** |
| Polyunsaturated Fatty Acids |  | -0.134(-0.192, -0.076) | **6.45×10^-6^** |
| Monounsaturated Fatty Acids |  | -0.055(-0.111, 0.001) | 5.36×10^-2^ |
| Saturated Fatty Acids |  | -0.071(-0.127, -0.014) | **1.38×10^-2^** |
| Linoleic Acid |  | -0.122(-0.179, -0.066) | **2.22×10^-5^** |
| Docosahexaenoic Acid |  | -0.105(-0.162, -0.048) | **3.24×10^-4^** |
| Amino acids |  |  |  |
| Alanine |  | -0.049(-0.103, 0.006) | 7.94×10^-2^ |
| Glutamine |  | 0.029(-0.025, 0.084) | 2.93×10^-1^ |
| Glycine |  | 0.085(0.025, 0.144) | **5.07×10^-3^** |
| Histidine |  | -0.052(-0.108, 0.004) | 7.10×10^-2^ |
| Total Concentration of Branched-Chain Amino Acids (Leucine + Isoleucine + Valine) |  | -0.148(-0.206, -0.089) | **8.40×10^-7^** |
| Isoleucine |  | -0.100(-0.157, -0.043) | **6.38×10^-4^** |
| Leucine |  | -0.139(-0.198, -0.080) | **4.15×10^-6^** |
| Valine |  | -0.154(-0.213, -0.095) | **2.55×10^-7^** |
| Phenylalanine |  | 0.027(-0.027, 0.080) | 3.27×10^-1^ |
| Tyrosine |  | -0.029(-0.083, 0.024) | 2.84×10^-1^ |
| Glycolysis related metabolites |  |  |  |
| Glucose |  | 0.053(0.001, 0.104) | **4.55×10^-2^** |
| Lactate |  | -0.026(-0.081, 0.029) | 3.55×10^-1^ |
| Pyruvate |  | 0.019(-0.038, 0.075) | 5.15×10^-1^ |
| Citrate |  | 0.085(0.041, 0.129) | **1.52×10^-4^** |
| Ketone bodies |  |  |  |
| 3-Hydroxybutyrate |  | 0.113(0.069, 0.157) | **5.05×10^-7^** |
| Acetate |  | 0.012(-0.032, 0.057) | 5.84×10^-1^ |
| Acetoacetate |  | 0.104(0.066, 0.142) | **8.77×10^-8^** |
| Acetone |  | 0.091(0.052, 0.130) | **5.10×10^-6^** |
| Fluid balance |  |  |  |
| Creatinine |  | 0.036(-0.009, 0.082) | 1.18×10^-1^ |
| Albumin |  | -0.054(-0.105, -0.003) | **3.64×10^-2^** |
| Glycoprotein Acetyls |  | 0.029(-0.028, 0.086) | 3.24×10^-1^ |
| Lipoprotein subclasses |  |  |  |
| Concentration of Chylomicrons and Extremely Large VLDL Particles |  | -0.058(-0.116, 0.000) | 5.02×10^-2^ |
| Total Lipids in Chylomicrons and Extremely Large VLDL |  | -0.060(-0.118, -0.003) | **3.86×10^-2^** |
| Phospholipids in Chylomicrons and Extremely Large VLDL |  | -0.055(-0.113, 0.003) | 6.33×10^-2^ |
| Cholesterol in Chylomicrons and Extremely Large VLDL |  | -0.057(-0.115, 0.001) | 5.38×10^-2^ |
| Cholesteryl Esters in Chylomicrons and Extremely Large VLDL |  | -0.058(-0.116, 0.001) | 5.31×10^-2^ |
| Free Cholesterol in Chylomicrons and Extremely Large VLDL |  | -0.055(-0.113, 0.003) | 6.31×10^-2^ |
| Triglycerides in Chylomicrons and Extremely Large VLDL |  | -0.060(-0.117, -0.003) | 4.06×10^-2^ |
| Concentration of Very Large VLDL Particles |  | -0.084(-0.143, -0.026) | **4.67×10^-3^** |
| Total Lipids in Very Large VLDL |  | -0.090(-0.147, -0.032) | **2.37×10^-3^** |
| Phospholipids in Very Large VLDL |  | -0.079(-0.137, -0.021) | **7.43×10^-3^** |
| Cholesterol in Very Large VLDL |  | -0.081(-0.139, -0.023) | **5.93×10^-3^** |
| Cholesteryl Esters in Very Large VLDL |  | -0.081(-0.139, -0.023) | **6.08×10^-3^** |
| Free Cholesterol in Very Large VLDL |  | -0.078(-0.136, -0.020) | **8.38×10^-3^** |
| Triglycerides in Very Large VLDL |  | -0.090(-0.148, -0.032) | **2.31×10^-3^** |
| Concentration of Large VLDL Particles |  | -0.094(-0.153, -0.036) | **1.51×10^-3^** |
| Total Lipids in Large VLDL |  | -0.106(-0.164, -0.049) | **2.84×10^-4^** |
| Phospholipids in Large VLDL |  | -0.091(-0.149, -0.033) | **2.19×10^-3^** |
| Cholesterol in Large VLDL |  | -0.091(-0.149, -0.033) | **2.04×10^-3^** |
| Cholesteryl Esters in Large VLDL |  | -0.086(-0.143, -0.028) | **3.63×10^-3^** |
| Free Cholesterol in Large VLDL |  | -0.092(-0.151, -0.034) | **1.86×10^-3^** |
| Triglycerides in Large VLDL |  | -0.113(-0.171, -0.055) | **1.31×10^-4^** |
| Concentration of Medium VLDL Particles |  | -0.091(-0.149, -0.033) | **2.14×10^-3^** |
| Total Lipids in Medium VLDL |  | -0.106(-0.163, -0.050) | **2.38×10^-4^** |
| Phospholipids in Medium VLDL |  | -0.089(-0.146, -0.031) | **2.50×10^-3^** |
| Cholesterol in Medium VLDL |  | -0.061(-0.119, -0.003) | **3.79×10^-2^** |
| Cholesteryl Esters in Medium VLDL |  | -0.043(-0.101, 0.015) | 1.47×10^-1^ |
| Free Cholesterol in Medium VLDL |  | -0.079(-0.137, -0.021) | **7.31×10^-3^** |
| Triglycerides in Medium VLDL |  | -0.116(-0.173, -0.058) | **7.64×10^-5^** |
| Concentration of Small VLDL Particles |  | -0.070(-0.127, -0.013) | **1.61×10^-2^** |
| Total Lipids in Small VLDL |  | -0.080(-0.137, -0.023) | **5.87×10^-3^** |
| Phospholipids in Small VLDL |  | -0.073(-0.131, -0.016) | **1.22×10^-2^** |
| Cholesterol in Small VLDL |  | -0.057(-0.114, 0.000) | 5.17×10^-2^ |
| Cholesteryl Esters in Small VLDL |  | -0.049(-0.106, 0.008) | 9.07×10^-2^ |
| Free Cholesterol in Small VLDL |  | -0.067(-0.124, -0.009) | **2.28×10^-2^** |
| Triglycerides in Small VLDL |  | -0.083(-0.141, -0.026) | **4.48×10^-3^** |
| Concentration of Very Small VLDL Particles |  | -0.028(-0.085, 0.029) | 3.31×10^-1^ |
| Total Lipids in Very Small VLDL |  | -0.028(-0.085, 0.029) | 3.36×10^-1^ |
| Phospholipids in Very Small VLDL |  | -0.013(-0.069, 0.044) | 6.62×10^-1^ |
| Cholesterol in Very Small VLDL |  | -0.027(-0.085, 0.031) | 3.58×10^-1^ |
| Cholesteryl Esters in Very Small VLDL |  | -0.029(-0.087, 0.029) | 3.30×10^-1^ |
| Free Cholesterol in Very Small VLDL |  | -0.021(-0.078, 0.036) | 4.70×10^-1^ |
| Triglycerides in Very Small VLDL |  | -0.033(-0.089, 0.024) | 2.57×10^-1^ |
| Concentration of IDL Particles |  | -0.052(-0.110, 0.006) | 7.88×10^-2^ |
| Total Lipids in IDL |  | -0.062(-0.120, -0.004) | **3.59×10^-2^** |
| Phospholipids in IDL |  | -0.045(-0.104, 0.014) | 1.33×10^-1^ |
| Cholesterol in IDL |  | -0.066(-0.125, -0.008) | **2.56×10^-2^** |
| Cholesteryl Esters in IDL |  | -0.073(-0.132, -0.015) | **1.43×10^-2^** |
| Free Cholesterol in IDL |  | -0.045(-0.104, 0.013) | 1.28×10^-1^ |
| Triglycerides in IDL |  | -0.020(-0.076, 0.037) | 4.91×10^-1^ |
| Concentration of Large LDL Particles |  | -0.053(-0.110, 0.005) | 7.24×10^-2^ |
| Total Lipids in Large LDL |  | -0.087(-0.144, -0.030) | **2.78×10^-3^** |
| Phospholipids in Large LDL |  | -0.082(-0.140, -0.025) | **5.22×10^-3^** |
| Cholesterol in Large LDL |  | -0.089(-0.146, -0.031) | **2.38×10^-3^** |
| Cholesteryl Esters in Large LDL |  | -0.092(-0.149, -0.035) | **1.64×10^-3^** |
| Free Cholesterol in Large LDL |  | -0.074(-0.132, -0.016) | **1.25×10^-2^** |
| Triglycerides in Large LDL |  | -0.025(-0.081, 0.032) | 3.89×10^-1^ |
| Concentration of Medium LDL Particles |  | -0.094(-0.152, -0.036) | **1.58×10^-3^** |
| Total Lipids in Medium LDL |  | -0.101(-0.157, -0.044) | **5.27×10^-4^** |
| Phospholipids in Medium LDL |  | -0.094(-0.152, -0.036) | **1.37×10^-3^** |
| Cholesterol in Medium LDL |  | -0.103(-0.161, -0.046) | **3.82×10^-4^** |
| Cholesteryl Esters in Medium LDL |  | -0.108(-0.165, -0.050) | **2.39×10^-4^** |
| Free Cholesterol in Medium LDL |  | -0.085(-0.142, -0.027) | **3.95×10^-3^** |
| Triglycerides in Medium LDL |  | -0.041(-0.098, 0.016) | 1.55×10^-1^ |
| Concentration of Small LDL Particles |  | -0.075(-0.133, -0.018) | **1.03×10^-2^** |
| Total Lipids in Small LDL |  | -0.080(-0.137, -0.023) | **5.81×10^-3^** |
| Phospholipids in Small LDL |  | -0.056(-0.112, 0.001) | 5.31×10^-2^ |
| Cholesterol in Small LDL |  | -0.087(-0.145, -0.030) | **2.93×10^-3^** |
| Cholesteryl Esters in Small LDL |  | -0.092(-0.150, -0.035) | **1.71×10^-3^** |
| Free Cholesterol in Small LDL |  | -0.063(-0.120, -0.006) | **2.90×10^-2^** |
| Triglycerides in Small LDL |  | -0.057(-0.114, 0.000) | 5.02×10^-2^ |
| Concentration of Very Large HDL Particles |  | 0.050(-0.010, 0.109) | 1.00×10^-1^ |
| Total Lipids in Very Large HDL |  | 0.068(0.009, 0.128) | **2.32×10^-2^** |
| Phospholipids in Very Large HDL |  | 0.072(0.013, 0.131) | **1.73×10^-2^** |
| Cholesterol in Very Large HDL |  | 0.065(0.006, 0.123) | **3.15×10^-2^** |
| Cholesteryl Esters in Very Large HDL |  | 0.060(0.000, 0.119) | **4.84×10^-2^** |
| Free Cholesterol in Very Large HDL |  | 0.077(0.020, 0.134) | **8.11×10^-3^** |
| Triglycerides in Very Large HDL |  | -0.037(-0.094, 0.019) | 1.97×10^-1^ |
| Concentration of Large HDL Particles |  | 0.028(-0.034, 0.089) | 3.75×10^-1^ |
| Total Lipids in Large HDL |  | 0.032(-0.029, 0.094) | 3.02×10^-1^ |
| Phospholipids in Large HDL |  | 0.028(-0.034, 0.090) | 3.72×10^-1^ |
| Cholesterol in Large HDL |  | 0.040(-0.021, 0.101) | 2.04×10^-1^ |
| Cholesteryl Esters in Large HDL |  | 0.039(-0.022, 0.100) | 2.09×10^-1^ |
| Free Cholesterol in Large HDL |  | 0.039(-0.022, 0.100) | 2.10×10^-1^ |
| Triglycerides in Large HDL |  | -0.043(-0.102, 0.015) | 1.48×10^-1^ |
| Concentration of Medium HDL Particles |  | -0.054(-0.115, 0.007) | 8.09×10^-2^ |
| Total Lipids in Medium HDL |  | -0.062(-0.122, -0.002) | **4.17×10^-2^** |
| Phospholipids in Medium HDL |  | -0.062(-0.122, -0.003) | **4.03×10^-2^** |
| Cholesterol in Medium HDL |  | -0.046(-0.106, 0.014) | 1.31×10^-1^ |
| Cholesteryl Esters in Medium HDL |  | -0.043(-0.103, 0.016) | 1.53×10^-1^ |
| Free Cholesterol in Medium HDL |  | -0.050(-0.111, 0.012) | 1.14×10^-1^ |
| Triglycerides in Medium HDL |  | -0.074(-0.131, -0.016) | **1.17×10^-2^** |
| Concentration of Small HDL Particles |  | -0.126(-0.183, -0.068) | **1.73×10^-5^** |
| Total Lipids in Small HDL |  | -0.125(-0.182, -0.068) | **1.49×10^-5^** |
| Phospholipids in Small HDL |  | -0.118(-0.175, -0.060) | **5.54×10^-5^** |
| Cholesterol in Small HDL |  | -0.119(-0.176, -0.063) | **3.64×10^-5^** |
| Cholesteryl Esters in Small HDL |  | -0.114(-0.171, -0.058) | **6.65×10^-5^** |
| Free Cholesterol in Small HDL |  | -0.112(-0.171, -0.054) | **1.55×10^-4^** |
| Triglycerides in Small HDL |  | -0.074(-0.132, -0.016) | **1.18×10^-2^** |
| Ratios |  |  |  |
| Ratio of triglycerides to phosphoglycerides |  | -0.062(-0.120, -0.004) | **3.49×10^-2^** |
| Ratio of apolipoprotein B to apolipoprotein A1 |  | -0.024(-0.080, 0.032) | 4.05×10^-1^ |
| Ratio of omega-3 fatty acids to total fatty acids |  | -0.076(-0.132, -0.020) | **8.07×10^-3^** |
| Ratio of omega-6 fatty acids to total fatty acids |  | 0.000(-0.052, 0.053) | 9.91×10^-1^ |
| Ratio of polyunsaturated fatty acids to total fatty acids |  | -0.029(-0.086, 0.029) | 3.25×10^-1^ |
| Ratio of monounsaturated fatty acids to total fatty acids |  | 0.008(-0.043, 0.059) | 7.63×10^-1^ |
| Ratio of saturated fatty acids to total fatty acids |  | 0.006(-0.039, 0.051) | 7.95×10^-1^ |
| Ratio of linoleic acid to total fatty acids |  | -0.062(-0.121, -0.003) | **3.91×10^-2^** |
| Ratio of docosahexaenoic acid to total fatty acids |  | -0.053(-0.108, 0.002) | 6.01×10^-2^ |
| Ratio of polyunsaturated fatty acids to monounsaturated fatty acids |  | -0.017(-0.073, 0.038) | 5.37×10^-1^ |
| Ratio of omega-6 fatty acids to omega-3 fatty acids |  | 0.087(0.032, 0.143) | **2.08×10^-3^** |
| Phospholipids to total lipids ratio in chylomicrons and extremely large VLDL |  | -0.012(-0.070, 0.046) | 6.86×10^-1^ |
| Cholesterol to total lipids ratio in chylomicrons and extremely large VLDL |  | 0.033(-0.021, 0.087) | 2.36×10^-1^ |
| Cholesteryl esters to total lipids ratio in chylomicrons and extremely large VLDL |  | 0.027(-0.029, 0.082) | 3.40×10^-1^ |
| Free cholesterol to total lipids ratio in chylomicrons and extremely large VLDL |  | 0.031(-0.020, 0.083) | 2.27×10^-1^ |
| Triglycerides to total lipids ratio in chylomicrons and extremely large VLDL |  | -0.026(-0.079, 0.027) | 3.34×10^-1^ |
| Phospholipids to total lipids ratio in very large VLDL |  | -0.051(-0.102, 0.001) | 5.59×10^-2^ |
| Cholesterol to total lipids ratio in very large VLDL |  | 0.085(0.033, 0.138) | **1.53×10^-3^** |
| Cholesteryl esters to total lipids ratio in very large VLDL |  | 0.078(0.025, 0.132) | **3.99×10^-3^** |
| Free cholesterol to total lipids ratio in very large VLDL |  | 0.074(0.032, 0.117) | **5.62×10^-4^** |
| Triglycerides to total lipids ratio in very large VLDL |  | -0.076(-0.130, -0.022) | **5.51×10^-3^** |
| Phospholipids to total lipids ratio in large VLDL |  | -0.062(-0.116, -0.009) | **2.11×10^-2^** |
| Cholesterol to total lipids ratio in large VLDL |  | 0.057(0.003, 0.111) | **3.89×10^-2^** |
| Cholesteryl esters to total lipids ratio in large VLDL |  | 0.070(0.015, 0.126) | **1.35×10^-2^** |
| Free cholesterol to total lipids ratio in large VLDL |  | 0.015(-0.039, 0.069) | 5.91×10^-1^ |
| Triglycerides to total lipids ratio in large VLDL |  | -0.016(-0.073, 0.040) | 5.67×10^-1^ |
| Phospholipids to total lipids ratio in medium VLDL |  | 0.024(-0.031, 0.080) | 3.87×10^-1^ |
| Cholesterol to total lipids ratio in medium VLDL |  | 0.060(0.003, 0.116) | **3.80×10^-2^** |
| Cholesteryl esters to total lipids ratio in medium VLDL |  | 0.064(0.007, 0.120) | **2.81×10^-2^** |
| Free cholesterol to total lipids ratio in medium VLDL |  | 0.050(-0.007, 0.106) | 8.33×10^-2^ |
| Triglycerides to total lipids ratio in medium VLDL |  | -0.057(-0.114, 0.000) | **4.84×10^-2^** |
| Phospholipids to total lipids ratio in small VLDL |  | 0.015(-0.043, 0.073) | 6.07×10^-1^ |
| Cholesterol to total lipids ratio in small VLDL |  | 0.035(-0.021, 0.091) | 2.18×10^-1^ |
| Cholesteryl esters to total lipids ratio in small VLDL |  | 0.047(-0.008, 0.102) | 9.59×10^-2^ |
| Free cholesterol to total lipids ratio in small VLDL |  | 0.015(-0.043, 0.073) | 6.18×10^-1^ |
| Triglycerides to total lipids ratio in small VLDL |  | -0.032(-0.089, 0.026) | 2.81×10^-1^ |
| Phospholipids to total lipids ratio in very small VLDL |  | 0.105(0.050, 0.161) | **1.89×10^-4^** |
| Cholesterol to total lipids ratio in very small VLDL |  | 0.005(-0.051, 0.061) | 8.59×10^-1^ |
| Cholesteryl esters to total lipids ratio in very small VLDL |  | 0.000(-0.056, 0.057) | 9.91×10^-1^ |
| Free cholesterol to total lipids ratio in very small VLDL |  | 0.032(-0.022, 0.087) | 2.39×10^-1^ |
| Triglycerides to total lipids ratio in very small VLDL |  | -0.028(-0.085, 0.029) | 3.37×10^-1^ |
| Phospholipids to total lipids ratio in IDL |  | 0.098(0.044, 0.152) | **3.62×10^-4^** |
| Cholesterol to total lipids ratio in IDL |  | -0.048(-0.100, 0.005) | 7.33×10^-2^ |
| Cholesteryl esters to total lipids ratio in IDL |  | -0.080(-0.133, -0.027) | **3.30×10^-3^** |
| Free cholesterol to total lipids ratio in IDL |  | 0.041(-0.011, 0.093) | 1.20×10^-1^ |
| Triglycerides to total lipids ratio in IDL |  | 0.024(-0.029, 0.078) | 3.78×10^-1^ |
| Phospholipids to total lipids ratio in large LDL |  | 0.046(-0.003, 0.095) | 6.42×10^-2^ |
| Cholesterol to total lipids ratio in large LDL |  | -0.041(-0.075, -0.008) | 1.53×10^-2^ |
| Cholesteryl esters to total lipids ratio in large LDL |  | -0.052(-0.079, -0.025) | **1.53×10^-4^** |
| Free cholesterol to total lipids ratio in large LDL |  | 0.010(-0.045, 0.064) | 7.29×10^-1^ |
| Triglycerides to total lipids ratio in large LDL |  | 0.045(-0.003, 0.094) | 6.73×10^-2^ |
| Phospholipids to total lipids ratio in medium LDL |  | 0.069(0.017, 0.121) | **9.31×10^-3^** |
| Cholesterol to total lipids ratio in medium LDL |  | -0.041(-0.074, -0.008) | **1.55×10^-2^** |
| Cholesteryl esters to total lipids ratio in medium LDL |  | -0.080(-0.119, -0.041) | **5.15×10^-5^** |
| Free cholesterol to total lipids ratio in medium LDL |  | 0.044(-0.012, 0.100) | 1.26×10^-1^ |
| Triglycerides to total lipids ratio in medium LDL |  | 0.039(-0.010, 0.087) | 1.15×10^-1^ |
| Phospholipids to total lipids ratio in small LDL |  | 0.112(0.061, 0.163) | **1.54×10^-5^** |
| Cholesterol to total lipids ratio in small LDL |  | -0.057(-0.092, -0.021) | **1.94×10^-3^** |
| Cholesteryl esters to total lipids ratio in small LDL |  | -0.090(-0.133, -0.047) | **4.39×10^-5^** |
| Free cholesterol to total lipids ratio in small LDL |  | 0.025(-0.030, 0.080) | 3.68×10^-1^ |
| Triglycerides to total lipids ratio in small LDL |  | -0.016(-0.071, 0.039) | 5.64×10^-1^ |
| Phospholipids to total lipids ratio in very large HDL |  | 0.100(0.039, 0.161) | **1.33×10^-3^** |
| Cholesterol to total lipids ratio in very large HDL |  | -0.065(-0.120, -0.010) | **2.15×10^-2^** |
| Cholesteryl esters to total lipids ratio in very large HDL |  | -0.024(-0.072, 0.025) | 3.38×10^-1^ |
| Free cholesterol to total lipids ratio in very large HDL |  | -0.061(-0.119, -0.002) | **4.24×10^-2^** |
| Triglycerides to total lipids ratio in very large HDL |  | -0.077(-0.135, -0.019) | **9.16×10^-3^** |
| Phospholipids to total lipids ratio in large HDL |  | -0.039(-0.094, 0.015) | 1.57×10^-1^ |
| Cholesterol to total lipids ratio in large HDL |  | 0.046(-0.009, 0.100) | 9.94×10^-2^ |
| Cholesteryl esters to total lipids ratio in large HDL |  | 0.037(-0.017, 0.090) | 1.81×10^-1^ |
| Free cholesterol to total lipids ratio in large HDL |  | 0.068(0.012, 0.124) | **1.76×10^-2^** |
| Triglycerides to total lipids ratio in large HDL |  | -0.047(-0.104, 0.010) | 1.04×10^-1^ |
| Phospholipids to total lipids ratio in medium HDL |  | 0.013(-0.043, 0.070) | 6.41×10^-1^ |
| Cholesterol to total lipids ratio in medium HDL |  | 0.012(-0.044, 0.068) | 6.72×10^-1^ |
| Cholesteryl esters to total lipids ratio in medium HDL |  | 0.021(-0.035, 0.076) | 4.65×10^-1^ |
| Free cholesterol to total lipids ratio in medium HDL |  | -0.031(-0.091, 0.030) | 3.23×10^-1^ |
| Triglycerides to total lipids ratio in medium HDL |  | -0.032(-0.089, 0.025) | 2.66×10^-1^ |
| Phospholipids to total lipids ratio in small HDL |  | 0.020(-0.037, 0.077) | 4.95×10^-1^ |
| Cholesterol to total lipids ratio in small HDL |  | 0.002(-0.053, 0.058) | 9.35×10^-1^ |
| Cholesteryl esters to total lipids ratio in small HDL |  | -0.006(-0.061, 0.050) | 8.37×10^-1^ |
| Free cholesterol to total lipids ratio in small HDL |  | 0.030(-0.027, 0.086) | 3.09×10^-1^ |
| Triglycerides to total lipids ratio in small HDL |  | -0.023(-0.080, 0.035) | 4.40×10^-1^ |

CI, confidence interval; LDL, Low Density Lipoprotein; HDL, High Density Lipoprotein; VLDL, Very Low Density Lipoprotein; IDL, Intermediate Density Lipoprotein. HR=exp(β).

Supplemental Table 3. Coefficients of Selected 24 metabolites in elastic net regularized logistic regression model after 10-fold cross validation, their coefficients in e-net regression model in training dataset, and their VIFs in testing dataset.

| Metabolites | Group | Subgroup | Coefficient | VIF |
| --- | --- | --- | --- | --- |
| Glutamine | Amino acids | NA | 0.117 | 1.42 |
| Histidine | Amino acids | NA | -0.104 | 1.34 |
| Leucine | Amino acids | Branched-chain amino acids | -0.0865 | 18.2 |
| Total Concentration of Branched-Chain Amino Acids | Amino acids | Branched-chain amino acids | -0.0737 | 19.9 |
| Tyrosine | Amino acids | Aromatic amino acids | 0.0339 | 1.77 |
| Alanine | Amino acids | NA | 0.0203 | 1.35 |
| Glycine | Amino acids | NA | -0.00930 | 1.24 |
| Linoleic Acid | Fatty acids | NA | -0.159 | 4.56 |
| Creatinine | Fluid balance | NA | -0.102 | 1.34 |
| Albumin | Fluid balance | NA | -0.0226 | 1.43 |
| Citrate | Glycolysis related metabolites | NA | 0.155 | 1.32 |
| Glucose | Glycolysis related metabolites | NA | 0.149 | 1.19 |
| Acetone | Ketone bodies | NA | 0.140 | 3.18 |
| 3-Hydroxybutyrate | Ketone bodies | NA | 0.0346 | 3.49 |
| Total Lipids in Small HDL | Lipoprotein subclasses | Small HDL (average diameter 8.7 nm) | -0.0112 | 27.6 |
| Cholesteryl Esters in Small HDL | Lipoprotein subclasses | Small HDL (average diameter 8.7 nm) | 0.00752 | 18.7 |
| Ratio of linoleic acid to total fatty acids | Ratio | NA | -0.256 | 3.61 |
| Cholesteryl esters to total lipids ratio in IDL | Ratio | NA | -0.107 | 6.76 |
| Phospholipids to total lipids ratio in small LDL | Ratio | NA | 0.0996 | 3.35 |
| Phospholipids to total lipids ratio in very large VLDL | Ratio | NA | -0.0547 | 1.68 |
| Free cholesterol to total lipids ratio in very small VLDL | Ratio | NA | 0.0251 | 5.20 |
| Free cholesterol to total lipids ratio in IDL | Ratio | NA | 0.0118 | 4.51 |
| Phospholipids to total lipids ratio in IDL | Ratio | NA | 0.00448 | 4.54 |
| Free cholesterol to total lipids ratio in very large VLDL | Ratio | NA | 0.00210 | 1.55 |

VIF, variance inflation factor; CI, confidence interval; LDL, Low Density Lipoprotein; HDL, High Density Lipoprotein; VLDL, Very Low Density Lipoprotein; IDL, Intermediate Density Lipoprotein; NA, Not Applicable.
